# Using Instructional Design, Analyze, Design, Develop, Implement, and Evaluate, to Develop e-Learning Modules to Disseminate Supported Employment for Community Behavioral Health Treatment Programs in New York State

**DOI:** 10.3389/fpubh.2018.00113

**Published:** 2018-05-07

**Authors:** Sapana R. Patel, Paul J. Margolies, Nancy H. Covell, Cristine Lipscomb, Lisa B. Dixon

**Affiliations:** ^1^The New York State Psychiatric Institute, Research Foundation for Mental Hygiene, New York, NY, United States; ^2^Department of Psychiatry, College of Physicians and Surgeons, Columbia University, New York, NY, United States; ^3^Intrac Inc., Instructional Design and Learning Strategy, Reno, NV, United States

**Keywords:** e-learning, supported employment, implementation science, instructional design, training

## Abstract

**Background:**

Implementation science lacks a systematic approach to the development of learning strategies for online training in evidence-based practices (EBPs) that takes the context of real-world practice into account. The field of instructional design offers ecologically valid and systematic processes to develop learning strategies for workforce development and performance support.

**Objective:**

This report describes the application of an instructional design framework—Analyze, Design, Develop, Implement, and Evaluate (ADDIE) model—in the development and evaluation of e-learning modules as one strategy among a multifaceted approach to the implementation of individual placement and support (IPS), a model of supported employment for community behavioral health treatment programs, in New York State.

**Methods:**

We applied quantitative and qualitative methods to develop and evaluate three IPS e-learning modules. Throughout the ADDIE process, we conducted formative and summative evaluations and identified determinants of implementation using the Consolidated Framework for Implementation Research (CFIR). Formative evaluations consisted of qualitative feedback received from recipients and providers during early pilot work. The summative evaluation consisted of levels 1 and 2 (reaction to the training, self-reported knowledge, and practice change) quantitative and qualitative data and was guided by the Kirkpatrick model for training evaluation.

**Results:**

Formative evaluation with key stakeholders identified a range of learning needs that informed the development of a pilot training program in IPS. Feedback on this pilot training program informed the design document of three e-learning modules on IPS: *Introduction to IPS, IPS Job development, and Using the IPS Employment Resource Book*. Each module was developed iteratively and provided an assessment of learning needs that informed successive modules. All modules were disseminated and evaluated through a learning management system. Summative evaluation revealed that learners rated the modules positively, and self-report of knowledge acquisition was high (mean range: 4.4–4.6 out of 5). About half of learners indicated that they would change their practice after watching the modules (range: 48–51%). All learners who completed the level 1 evaluation demonstrated 80% or better mastery of knowledge on the level 2 evaluation embedded in each module. The CFIR was used to identify implementation barriers and facilitators among the evaluation data which facilitated planning for subsequent implementation support activities in the IPS initiative.

**Conclusion:**

Instructional design approaches such as ADDIE may offer implementation scientists and practitioners a flexible and systematic approach for the development of e-learning modules as a single component or one strategy in a multifaceted approach for training in EBPs.

## Background and Rationale for Educational Activity

A recent report by the Institute of Medicine *Best Care at a Lower Cost: The Path to Continuously Learning Health Care in America* (1), reported, “Achieving higher quality care at lower cost will require fundamental commitments to the incentives, culture, and leadership that foster continuous learning, as the lessons from research and each care experience are systematically captured, assessed, and translated into reliable care.” Central to the translation from research to practice and reliable care is training health-care providers in evidence-based practices (EBPs). In behavioral health care, training in EBPs often involves developing new clinical competencies. This training should take into account the context and needs of the practice community as well as strategies to facilitate adoption and implementation (2). Increasingly, training utilizes online modalities to expand its reach and efficiency, digital media to promote active engagement, shorter learning sessions to foster knowledge retention, and methods to demonstrate and practice skills that can be applied in the workplace (3).

**Figure 1:**
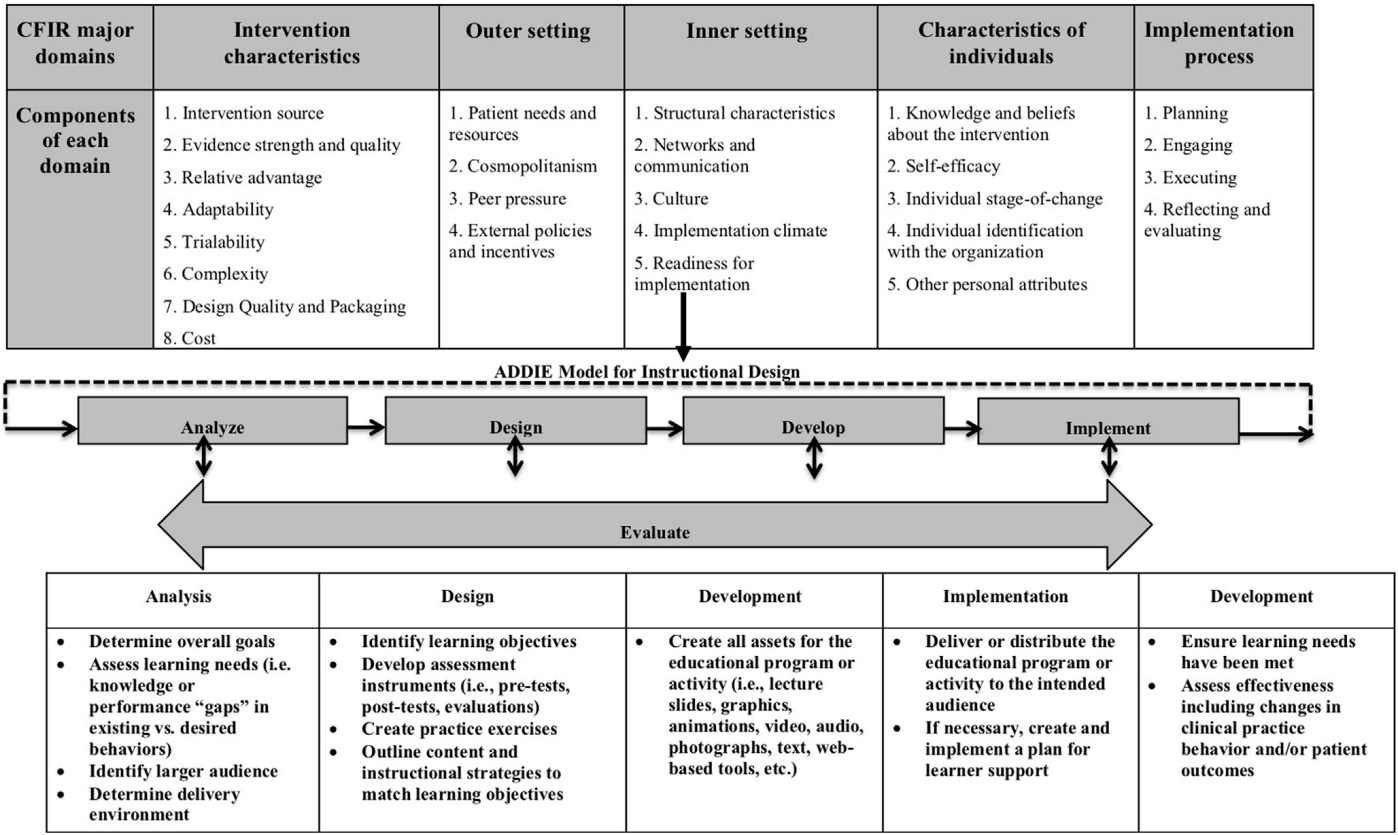
Using an instructional design framework [Analyze, Design, Develop, Implement, and Evaluate (ADDIE)] as a systematic process to develop training in an implementation framework [Consolidated Framework for Implementation Research (CFIR)].

Implementation science, a field dedicated to understanding targeted dissemination and implementation of EBPs and the use of strategies to improve adoption in community health-care settings, has guided the work of translating research to practice. Numerous frameworks in implementation science provide a menu of constructs that have been associated with effective implementation. Damschroder (Figure 1) (4) combined 19 published implementation theories into the Consolidated Framework for Implementation Research (CFIR). The CFIR provides a menu of constructs that have been associated with effective implementation. The framework is organized into five domains: *intervention characteristics, inner setting, outer setting, characteristics of individuals, and process*. Under the inner setting domain, one key construct under the component *readiness for implementation* is *access to knowledge and information*. *Access to knowledge and information* is defined as the ease of access to digestible information and knowledge about the practice and how to incorporate it into work tasks. This is the function of training. It is purported that when timely on-the-job training is available, implementation is more likely to be successful (5).

Although training is an important determinant of successful implementation (5), the field of implementation science lacks systematic approaches for the development of training that takes into account the learners’ needs, context, and optimal modalities for learning. Training in EBPs and their evaluation has been identified as a priority item on the National Institutes of Health research agenda (Program Announcement in Dissemination and Implementation Research in Health; https://grants.nih.gov/grants/guide/pa-files/PAR-16-238.html) and a commonly used implementation strategy in implementation practice and research (6). Intervention or EBP developers are not likely to have expertise in instructional design and may miss the mark of engaging busy practitioners in training for several reasons. First, didactic approaches may not take into account the level of interest or needs of the practitioners. Second, traditional training approaches may not consider organizational factors (i.e., time available for provider training) also key to successful implementation (7). Third, how individuals learn and process information is evolving given our access to the Internet and technology. The field of implementation science may benefit from an ecologically valid approach to the development of learning experiences for training health-care practitioners.

Recent reports have pointed to the utility of instructional design in the dissemination and implementation of EBPs in behavioral health (8, 9). The field of instructional design offers one model, Analyze, Design, Develop, Implement and Evaluate (ADDIE) (10), that takes into account learning theory, the learner’s needs and environment, and approaches to training practitioners in EBPs. The foundations of ADDIE are traced back to World War II when the U.S. military developed strategies for rapidly training people to perform complex technical tasks. The ADDIE model is used in creating a teaching curriculum or a training that is geared toward producing specific learning outcomes and behavioral changes. It provides a systematic approach to the analysis of learning needs, the design and development of a curriculum, and the implementation and initial evaluation of a training program (11, 12). This model of developing training programs is particularly useful if the focus of the program is targeted toward changing participant behavior and improving performance. ADDIE is increasingly being adopted in industries such as health care (13). Recent studies have successfully adopted the ADDIE model to improve patient safety, procedural competency, and disaster simulation (14, 15). It has also been effectively used in medical training and education to change practice behaviors in the management of various medical conditions (16–18).

The options for delivery and modalities used in training (e.g., mobile devices, webcasts, and podcasts) have expanded significantly in the last decade. With online learning technology, there is an opportunity to reach learners anytime and anywhere to provide performance support. One such example of an online learning technology is e-learning modules. e-Learning modules are self-paced lessons that enable the learner to read text, listen to narrated content, observe video scenarios, and respond to questions or prompts, in a multimedia format designed to maximize engagement and retention. Learning management systems (LMSs) host e-learning modules and capture learning metrics and performance. Learning analytics provided by LMSs enable the ability to track individual and group performance which may be used to provide feedback and support continuous learning in large systems of care.

In this report, we provide an example of the application of ADDIE in the development and evaluation of e-learning modules as one strategy among a multifaceted approach to the dissemination and implementation of the individual placement and support (IPS) model of supported employment in community treatment programs in New York State (NYS). Specifically, we (1) describe the application of an instructional design framework, ADDIE, in the iterative development of e-learning modules for IPS; (2) conduct a large-scale dissemination of the IPS e-learning modules throughout the state using an LMS; (3) evaluate learner reaction, self-reported knowledge, and practice change after IPS e-learning modules; (4) identify key barriers and facilitators to future IPS implementation using formative and summative ADDIE evaluation data and the CFIR.

## Pedagogical Frameworks

We used three frameworks to guide the process of developing e-learning modules (ADDIE), identify determinants of future training and implementation (CFIR), and evaluate the IPS e-learning modules [Kirkpatrick model (19)]. The ADDIE model consists of five phases, beginning with identifying key stakeholder needs, educational goals, and optimal methods of content delivery (analysis). This information was used to establish a design document for the training (design) that is vetted by key stakeholders prior to building the e-learning modules (development). After iterative refinement, e-learning modules were disseminated and evaluated using the Kirkpatrick model for training evaluation (19) (implementation/evaluation). Results from the formative and summative evaluations conducted during the ADDIE process, identified barriers/facilitators to implementation using CFIR domains (Figure 1). Doing so allowed the IPS team to iteratively ensure sufficient attention to contextual variables, align with the larger conceptual and empirical implementation literature (9, 20) as well as select strategies to build a multifaceted approach to IPS implementation.

## Learning Environment

In November 2007, the NYS Office of Mental Health (OMH) and the Department of Psychiatry, Columbia University, established the Center for Practice Innovations (CPI) at Columbia Psychiatry and New York State Psychiatric Institute to promote the widespread use of EBPs throughout NYS. CPI uses innovative approaches to build stakeholder collaborations, develop and maintain providers’ expertise, and build agency infrastructures that support implementing and sustaining these EBPs. CPI works with OMH to identify and involve consumer, family, provider, and scientific-academic organizations as partners in supporting the goals of OMH and CPI. CPI’s initial charge was to provide training for the NYS behavioral health-care workforce. Given the size and geographical dispersion of this workforce, CPI turned to distance-learning technologies and e-learning modules (21, 22). Distance technologies may offer cost-effective alternatives to typical training methods, and some evidence suggests that such technologies are at least as effective as a face-to-face training (21). CPI has collaborated with key stakeholders and content experts to create more than 100 e-learning modules to provide training for its initiatives. CPI’s online modules and resources require the use of an online learning platform, an LMS, that facilitates access to online training, event registration, and resource libraries for each initiative.

One of these initiatives, IPS, provides training and implementation support in an evidence-based approach to supported employment (23). Rates of competitive employment were low across NYS, with a competitive employment rate of 9.2% in 2011 prior to systematic IPS implementation (Patient Characteristics Survey data, 2011 obtained from https://www.omh.ny.gov/omhweb/statistics/). In response, OMH leadership identified supported employment as a key service in personalized recovery oriented services (PROS) programs, a comprehensive model that integrates rehabilitation, treatment, and support services for people with serious mental illness. The number of PROS programs in New York has increased significantly over the past decade: in 2017, 86 programs were serving 10,500 individuals. In order to reach these 86 programs statewide, the IPS initiative developed a series of three e-learning modules: *Introduction to IPS, IPS Job Development*, and *Using the IPS Employment Resource Book*. The module development team included an instructional designer, subject matter experts (SMEs), course developers, and a project manager.

## Pedagogical Format: E-Learning Module Design Using Addie

### Analysis: Learning Objectives

In the analysis phase, the instructional problem was clarified, the instructional goals and objectives were established, and the learner’s environment, existing knowledge, and skills were identified. The module development team engaged in a discussion to identify the instructional problem and understand the expectations for performance after completing the modules. Because IPS had not been previously implemented in NYS, it was expected that learners’ existing knowledge and skills of IPS would be minimal. Formative evaluation *via* preliminary discussions with agency administrators, employment supervisors, and employment staff members in PROS programs included questions about learners’ experiences with and opinions about traditional vocational rehabilitation methods, attitudes about IPS principles (i.e., zero exclusion), awareness of or experiences with IPS, and expectations and attitudes about the likelihood of program recipients in their programs working competitively. These discussions revealed several needs: lack of understanding of the evidence for IPS (*CFIR: intervention*), discomfort with some IPS principles which are inconsistent with traditional approaches to vocational rehabilitation (*characteristics of individuals*), lack of knowledge about the specific skills and tasks involved in the model (*characteristics of individuals*), lack of familiarity with how to do job development and why it is important (*characteristics of individuals*), and the lack of tools that can be used in real-time meetings with potential employers (*implementation process*). These data informed the development of a curriculum for a pilot training program in IPS that consisted of in-person training, webinars, and on-site technical assistance. Through this pilot process, observations were made about learners’ strengths and additional training needs, and the PROS program environment. In addition, program recipients’ (adults diagnosed with serious mental illness, living in the community, many with histories of hospitalizations and treatment) employment needs (e.g., consistent with individuals’ personal strengths and interests), part-time for many, easily accessible with public transportation (*outer setting*) supported another cycle of modifications to the IPS curriculum and informed decisions about pedagogical format. As the initiative required scalability across the state of New York, it was determined that e-learning modules would be an important resource-efficient implementation strategy.

### Design

The design phase established learning objectives, exercises, content, lesson planning, and media selection *via* a design document, which served as the blueprint for building the training program. The instructional designer gathered feedback from the analysis phase and resources on the topic provided by SMEs (e.g., books, research publications, information available online) and identified content to support the learning objectives (Table 1) for all three IPS e-learning modules. The module development team designed a 10-item knowledge quiz and a 10-item level 1 reaction survey consisting of both closed- and open-ended questions. Iteratively, the instructional designer presented design documents for review and feedback from the module development team. An example design document for the IPS Job Development module is provided in the Figure S1 in Supplementary Material.

**Table 1:** Learning objectives for individual placement and support (IPS) modules.

Introduction to IPS	Learn about the historical context for IPS Learn about why employment is important Learn core practitioner skills that follow fundamentals of IPS and its implementation
IPS Job Development Module	Understand the importance of job development and the employment specialist role Learn about the role of the treatment team and how each member can support job development Learn about how to coach consumers as they meet employers, build networks, discuss disclosure
Using the IPS Employment Resource Book	Learn about the Employment Resource Book and its usefulness for consumers, family members, and providers

### Development

During the development phase, the course developer received the reviewed design document and used an authoring tool software to create multimedia e-learning modules according to the design document. During this phase, the IPS modules were animated using video, graphics with narration, knowledge checks, and photographs. Formative evaluation from the analysis phase led to the development of a tool, the Employment Resource Book (24), that could be utilized by key stakeholders (providers, supervisors, and recipients) during any phase of employment (e.g., considering work, actively seeking employment, maintaining employment), and one module was developed to provide guidance about using this resource. The IPS training was built into three short e-learning modules to reflect learners’ time availability and attention span during the workday, then tested in prototype with the module development team and revised.

### Implementation

During the implementation phase, e-learning modules were uploaded to the CPI LMS for usability testing. During usability testing, the module’s functionality is evaluated prior to training implementation. For example, the module development team tested whether videos play and navigation works (e.g., next buttons and links to additional resources) on a variety of web browsers and devices. Feedback from the usability testing phase is used to fix errors in navigation and improve user experience (25). After usability testing issues were addressed, the modules were ready for implementation.

When the IPS initiative began, the NYS-OMH Rehabilitation Services Unit sent an official email communication strongly encouraging PROS program providers and supervisors to participate in the training offered by the CPI IPS initiative. Further, each PROS program supervisor received an email, alerting them that the new IPS e-learning module was available in CPI’s LMS. Through the LMS, PROS program participation in the modules was tracked, and completion could be monitored by PROS programs and NYS-OMH.

### Evaluation

We applied quantitative and qualitative methods as part of formative and summative evaluation in the ADDIE process. Formative evaluations consisted of qualitative feedback received from recipients and providers during early pilot work, which identified training needs. The summative evaluation consisted of quantitative and qualitative data and was guided by the Kirkpatrick model for training evaluation (19). The four levels of evaluation are (1) the reaction of the learner about the training experience, (2) the learner’s resulting learning and increase in knowledge from the training experience, (3) the learner’s behavioral change and improvement after applying the skills on the job, and (4) the results or effects that the learner’s performance has on care provided. For this report, we focus on the first two levels, specifically, the level 1—reaction of the learner including training experience, self-reported knowledge acquisition, and self-reported practice change through a survey and level 2—resulting knowledge through post-module quizzes.

To keep the learner experience seamless, a decision was made to embed the knowledge quiz, assessing knowledge of IPS model-related concepts, skills, and tools, within each module. In order for the module to be marked as completed, learners are required to answer at least 80% of the knowledge items correctly, which satisfies continuing education accreditation requirements. Learners are able to retake the quiz as many times as needed to meet this criterion score. Once the module is completed, the learner is prompted to complete the level 1 survey. The level 1 reaction survey was based on learning objectives set forth in each e-learning module, accreditation requirements, and example questions from Kirkpatrick level 1 (19). Questions included rating the module overall, if it met stated learning objectives, if the information presented was new to the learner, and questions about module-specific self-reported knowledge and practice change. In addition, three open-ended questions were included: What could we improve? What do you like the most about this module? and Where do you think you might use what you learned in this module?

The NYS Psychiatric Institute Institutional Review Board determined that this evaluation did not meet the definition of human subject research.

### Analysis

Using IBM© SPSS© Statistics Version 24, we applied descriptive statistics to quantitative level 1 summative evaluation data. For the qualitative formative and summative evaluation data, we employed a thematic analysis to identify themes within the open-ended question data (26). Two coders reviewed the open-ended question data independently to identify codes and develop an initial code list. The coders combined codes into overarching themes and met to review and label them. Coders met twice to discuss discrepancies and achieve consensus on key barriers and facilitators within the CFIR framework. We report on those themes that were raised by at least 10% of the sample.

## Results

We describe the inputs and outputs during each phase of IPS module development using ADDIE in Table 2. Formative evaluation during each stage of ADDIE allowed for the iterative revision of the content for each module and the identification of needs for subsequent modules. Feedback received from the evaluation of the first module led to the development of the second module (i.e., desire to learn more about job development) and to the development of the Employment Resource Book including the associated third module (i.e., desire to be better equipped to deal with common challenges).

**Table 2:** Using Analyze, Design, Develop, Implement, and Evaluate (ADDIE) to develop individual placement and support modules.

ADDIE model	Inputs	Outputs
Analysis	Preliminary discussions with personalized recovery oriented service program providers, recipients, Office of Mental Health staff	Learning needs data
		Pilot curriculum and training program
		Revised learning objectives and format to scale training

Design	Learning objectives, development of evaluation, and media selection	Design document
	SME and module development team of design document	Revised design document

Development	Authoring tool applied to design document to develop multimedia training	Multimedia e-learning module
	Module development and learning management system team review of module	

Implementation	Assessment of technical fit and specifications	Refinement of course
	Pilot launch: usability testing	

Evaluation	Evaluation design and monthly review of results	Level 1 and 2 evaluation results
		Barriers and facilitators to future implementation beyond e-learning

Summative evaluation examined the impact of the IPS training modules and assisted in the identification of barriers and facilitators for IPS implementation in the future. Table 3 summarizes level 1 evaluation data for all three IPS modules. Learners’ background and experience varied considerably across programs. Many were rehabilitation counselors, social workers, and some had non-behavioral health backgrounds. Learners rated all three modules highly (mean range: 4.4–4.5 out of 5). Learners also indicated that the modules presented new information and met their stated learning objectives (mean range: 4.3–4.4 out of 5). Similarly, learners’ self-report of knowledge acquisition was high (mean range: 4.4–4.6 out of 5). About half of learners indicated that they would change their practice after watching the modules (range: 48–51%). All learners who completed the level 1 evaluation demonstrated 80% or better mastery of knowledge on the level 2 evaluation embedded in each module.

**Table 3:** Level 1 data from all three individual placement and support (IPS) modules.

	*Introduction to IPS^a^*(M, SD)	*IPS Job Development^b^*(M, SD)	*Using the IPS Employment Resource Book^c^*(M, SD)
**Reaction**			
I would rate this training (with five stars being the best)	4.5 (0.74)	4.4 (0.77)	4.5 (0.73)
The online module met its stated objectives (1 = very inadequately to 5 = considerably)	4.4 (0.80)	4.3 (0.76)	4.4 (0.60)
The module included information that was new to me^d^	4.2 (0.88)	4.2 (0.91)	4.3 (0.74)

**Self-reported knowledge^d^**			
*IPS: Introduction to the IPS Model of Supported Employment*			
As a result of this online module, I better understand the importance of employment for persons with mental illness	4.5 (0.80)		
As a result of this online module, I better understand the rationale for and fundamentals of IPS	4.5 (0.76)		
As a result of this online module, I better understand core practitioner skills and how to implement IPS	4.5 (0.73)		

**IPS: Job Development**			
As a result of this online module, I better understand the importance of job development		4.4 (0.75)	
As a result of this online module, I better understand the importance of the employment specialist role		4.5 (0.77)	
As a result of this online module, I better understand how to support job development across the treatment team		4.4 (0.76)	

**IPS: Using the Employment Resource Book**			
As a result of this training, I better understand how to access the Employment Resources Book			4.6 (0.60)
As a result of this training, I better understand how to use the book for guidance and direction concerning consumers’ employment goals			4.6 (0.61)

**Self-reported practice change**	***N*(%)**	***N*(%)**	***N*(%)**
This activity validated my current practice; no changes will be made	49%	49%	52%
Change the management and/or treatment of my patients/clients	31%	28%	27%
Create/revise protocols, policies, and/or procedures	20%	20%	21%

*^a^N = 523*.

*^b^N = 312*.

*^c^N = 127*.

*^d^Likert scale: 1-strongly disagree to 5-strongly agree*.

Open-ended question themes and related CFIR domains from these e-learning modules helped identify additional implementation support needs to be addressed by the multifaceted approach to implementing IPS (i.e., statewide webinars, regional online meetings focusing on special topics such as IPS fidelity and supervision, an IPS library with tools to help IPS implementation, and individualized program consultations that focus on addressing implementation challenges and enhancing provider competence). Themes from the open-ended questions for all three IPS modules are described using the CFIR in Table 4. These themes related to three CFIR domains: outer setting, inner setting, and implementation process. They provided information on how the modules were acceptable, what the future learning needs are, and how the information learned will be used in everyday practice.

**Table 4:** Themes and CFIR domains from level 1 survey open-ended questions for individual placement and support (IPS) modules.

Open-ended questions	Themes		
	*Introduction to IPS*	*IPS Job Development*	*Using the IPS Employment Resource Book*
What could we improve?	More on common challenges (symptoms developing, substance use, disclosure, failed attempts) (*outer setting*)	More on connecting with potential employers (*outer setting*)	Using materials in groups (*inner setting*)
	How to do job development (*inner setting*)	More real-world examples and scenarios (*outer setting*)	

What do you like the most about this module?	Learning to be more person-centered, person-driven, de-stigmatizing (*inner setting*)	Breakdown of three visits with potential employers (*inner setting*)	Workbook (*inner setting*)
	How to do rapid job search (*inner setting*)		Breaking down steps of job explorations (*inner setting*)

Where do you think you might use what you learned in this module?	One-on-one with clients (*implementation process*)	Engaging in job development in the community (*implementation process*)	With clients/king work (*implementation process*)
	Supervision (*implementation process*)	During supervision and groups (*implementation process*)	Supervision (*implementation process*)
			Special populations (veterans, those with criminal background) (*implementation process*)

## Discussion

This report provides one example of how an instructional design approach may be applied to the development of e-learning modules as one strategy in a multifaceted approach to the implementation of IPS supported employment for community program providers in a large state public behavioral health system. Through iterative development, we applied the ADDIE model to develop a series of e-learning modules for IPS. Using an LMS, these modules were disseminated and evaluated by PROS program providers throughout NY state. Results from both level 1 and level 2 evaluations indicate that the ADDIE model was successful in improving practitioner knowledge. In addition, learners received the e-learning modules favorably, rating them highly overall and noting that they met stated learning objectives and presented new information. Throughout the development process, data from the e-learning modules were described using the CFIR to identify needs that led to additional e-learning modules as well as strategies for subsequent implementation supports through a learning collaborative statewide (27).

The ADDIE model and CFIR were used as complementary approaches in the development of e-learning resources for training providers in an EBP. Our experience in this process produced several lessons learned and recommendations for implementation researchers and practitioners. The analysis phase of the ADDIE model required assessment of multistakeholder needs and context early on in the process of developing training. We recommend taking the time to assess and include end users and recipients to shape and increase the ecological validity of the training. In addition, the use of the CFIR domains allowed us to anticipate barriers and map future implementation strategies. During the design process, the establishment of clear and measureable learning objectives was important and facilitated focus and evaluation of knowledge and skill acquisition. We recommend the *a priori* assembly of e-learning module development teams to work with the instructional designer and establish an efficient process for the review of training content and format through weekly iterative review meetings during the design and development stages. Although the ADDIE process points to the introduction of the learning platform (e.g., website, LMS) at the implementation stage, we would recommend that the team with technical expertise (i.e., in our case, the courseware developers and LMS administrators) be introduced earlier in the process during the development stage. This is crucial to the feasibility and usability of the end product. Once implemented, we recommend a scheduled monthly review of the evaluation data that is being collected as learners participate in the e-leaning modules. This information will identify any needed revisions to the training content, the need for future content development, and barriers and facilitators for future implementation.

This article reports on the development of e-learning modules that were one part of a larger implementation effort in a state system. This implementation was not a part of a rigorous research evaluation. Limitations of this report include inability to formally assess pre–post knowledge, practice and readiness for IPS implementation using validated scales based on accepted standard in the literature, variation in sample sizes for the e-learning modules precluding examination of a stable cohort of learners over time, and the inability to directly assess the specific impact of these e-learning modules on employment outcomes apart from other elements of the entire initiative. Notably, only half of the providers who completed the evaluation noted an intention to change their practice, and we did not have the capacity to assess practice change at the individual provider level at this stage of IPS implementation (level 3). However, in our subsequent work (27), program fidelity assessments using established measures demonstrated improvement over time, suggesting that level 3 provider practice change and fidelity self-assessed by program sites are shown to be associated with higher employment rates (level 4), which are sustained over time (28). Future research may focus on more rigorous evaluation of knowledge, practice change, mixed-method assessment of how the content from e-learning modules influences practice, and the essential role of care recipients in helping to design training within implementation efforts.

From adoption to sustainability, implementation science focuses on strategies to promote the systematic uptake of research findings into routine practice. Successful implementation relies on iterative, interacting activities that follow a systematic process for strategy development. In the case of training as an implementation strategy, instructional design offers a systematic and iterative process. First, it applies instructional theory to the development of training regardless of subject matter. Second, it identifies fundamental elements of the learners’ needs and real-world setting factors in addition to the EBP being implemented. Third, it creates accountability to align training content with measurable learning objectives and assesses learner knowledge and skill acquisition based on content. Lastly, it engages multimedia novel approaches in the development of educational and training resources.

Compared to more intensive approaches to training and workforce development, the development of e-learning modules informed by an instructional design approach provides implementation science the opportunity to scale and support training at the level of knowledge and skill acquisition for a range of EBPs. These modules can be used either as stand-alone or as part of blended learning activities and implementation supports as in the IPS initiative. Another example, in the case of complex, multi-component intervention or model of care, is CPI’s work with Assertive Community Treatment, where instructional design is used to develop e-learning modules as a first step in a blended learning training curriculum for practitioners in a state system (29). As such, there is increasing interest in examining the effect of an instructional design approach to training in behavioral health, especially for large systems of care.

## Conclusion

Instructional design approaches such as ADDIE may offer implementation scientists and practitioners a flexible and systematic guideline for the development of e-learning modules as a single component or one strategy in a multifaceted approach for training practitioners in EBPs. In this way, this approach facilitates the translation between science to practice that takes into account the context of the learner and leverages technology for expanded reach, both promising approaches for workforce development and a learning health-care system (1, 30).

## Author Contributions

SP, CL, and LD conceived the study and conceptual framework. SP and NC managed data and analyses. SP, PM, NC, and CL contributed to writing the manuscript with feedback and supervision from LD.

## Conflict of Interest Statement

The authors declare that the research was conducted in the absence of any commercial or financial relationships that could be construed as a potential conflict of interest. The reviewer AR and the handling Editor declared their shared affiliation.

## Supplementary Material

Figure S1 IPS job development design document.
