# Supplementary figures and images for: Using Instructional Design, Analyze, Design, Develop, Implement, and Evaluate, to Develop e-Learning Modules to Disseminate Supported Employment for Community Behavioral Health Treatment Programs in New York State
Source: Front Public Health. 2018 May 7;6:113. doi: 10.3389/fpubh.2018.00113 (PMC5949337; doi:10.3389/fpubh.2018.00113)

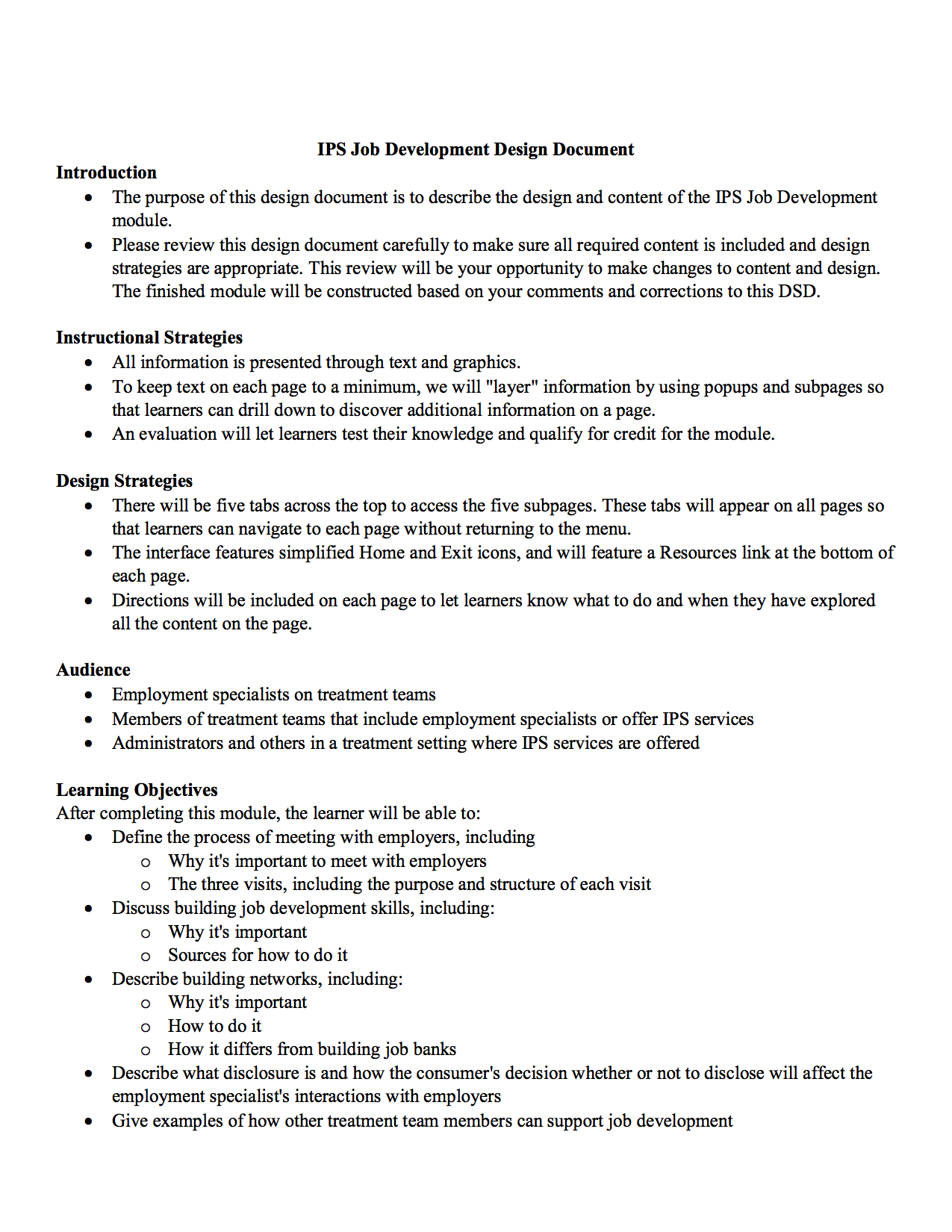

Supplement: Figure S1 — IPS job development design document. [file Image_1.tiff]
